# Molecular Typing of Pathogenic *Leptospira* Serogroup Icterohaemorrhagiae Strains Circulating in China during the Past 50 Years

**DOI:** 10.1371/journal.pntd.0003762

**Published:** 2015-05-19

**Authors:** Cuicai Zhang, Huimian Yang, Xiuwen Li, Zhiqiang Cao, Haijian Zhou, Linzi Zeng, Jianmin Xu, Yinghua Xu, Yung-Fu Chang, Xiaokui Guo, Yongzhang Zhu, Xiugao Jiang

**Affiliations:** 1 State Key Laboratory for Infectious Disease Prevention and Control, National Institute for Communicable Disease Control and Prevention, Chinese Centre for Disease Control and Prevention, Beijing, People's Republic of China; 2 Collaborative Innovation Center for Diagnosis and Treatment of Infectious Diseases, Hangzhou, People’s Republic of China; 3 Mentougou District Center for Disease Control and Prevention, Beijing, People's Republic of China; 4 SiChuan Province Center for Disease Control and Prevention, Chengdu, People's Republic of China; 5 JiangXi Province Center for Disease Control and Prevention, Nanchang, People's Republic of China; 6 Key Laboratory of the Ministry of Health for Research on Quality and Standardization of Biotech Products, National Institutes of Food and Drug Control, Beijing, People's Republic of China; 7 Department of Population Medicine and Diagnostic Sciences, College of Veterinary Medicine, Cornell University, Ithaca, New York, United States of America; 8 Department of Microbiology and Immunology, Institute of Medical Science, Shanghai Jiao Tong University School of Medicine, Shanghai, People's Republic of China; Fondation Raoul Follereau, FRANCE

## Abstract

**Background:**

Leptospirosis is one of the most important neglected tropical infectious diseases worldwide. Icterohaemorrhagiae has been throughout recent history, and still is, the predominant serogroup of this pathogen in China. However, very little in detail is known about the serovars or genotypes of this serogroup.

**Methodology/Principal Findings:**

In this study, 120 epidemic strains from five geographically diverse regions in China collected over a 50 year period (1958~2008), and 8 international reference strains characterized by 16S rRNA sequencing and MLST analysis. 115, 11 and 2 strains were identified as *L*. *interrogans*, *L*. *borgpetersenii*, and *L*. *kirschneri*, respectively. 17 different STs were identified including 69 ST1 strains, 18 ST17, 18 ST128, 9 ST143 and 2 ST209. The remaining 12 strains belonged to 12 different STs. eBURST analysis demonstrated that, among the clonal complexes isolated (CCs), CC1 accounted for 73.3% (88/120) strains representing three STs: ST1, ST128 and ST98. ST1 was the most likely ancestral strain of this CC, followed by singleton CC17 (17/120) and CC143 (11/120). Further analysis of adding 116 serogroup Icterohaemorrhagiae strains in the MLST database and studies previously described using global eBURST analysis and MST dendrogram revealed relatively similar ST clustering patterns with five main CCs and 8 singletons among these 244 strains. CC17 was found to be the most prevalent clone of pathogenic *Leptospira* circulating worldwide. This is the first time, to our knowledge, that ST1 and ST17 strains were distributed among 4 distinct serovars, indicating a highly complicated relationship between serovars and STs.

**Conclusions/Significance:**

Our studies demonstrated a high level of genetic diversity in the serogroup Icterohaemorrhagiae strains. Distinct from ST17 or ST37 circulating elsewhere, ST1 included in CC1, has over the past 50 years or so, proven to be the most prevalent ST of pathogenic leptospires isolated in China. Moreover, the complicated relationship between STs and serovars indicates an urgent need to develop an improved scheme for *Leptospira* serotyping.

## Author Summary

Leptospirosis, caused by pathogenic *Leptospira spp*, is a globally widespread zoonosis. In this study, our focusing on serogroup Icterohaemorrhagiae strains of *Leptospira* using MLST as a tool for phylogenetic analysis that has led to a better understanding of evolution of *Leptospira*. This totally consisted of 120 epidemic strains from five geographically diverse regions were isolated over the past 50 years in China and 8 strains from seven different countries. 17 STs were identified in these 128 strains by MLST analysis. Adding 116 serogroup Icterohaemorrhagiae in the *Leptospira* MLST database and studies previously described, 22 STs were identified in the 244 isolates. The genetic diversity of *Leptospira* belonging to serogroup Icterohaemorrhagiae from China was generally different from that of isolates elsewhere. Results of the 16S rRNA sequencing typing and MLST genotyping method were nearly consistent. Here, MLST revealed the high diversity of STs among the serogroup Icterohaemorrhagiae strains in China. Our present study provides a blueprint for further phylogenetic research. More convenient molecular techniques have to be developed to identify and characterize *Leptospira* species and STs.

## Introduction

Leptospirosis, caused by pathogenic *Leptospira* species, is emerging as one of the most widespread zoonosis with an estimated global burden of more than 500,000 cases of severe human leptospirosis and 100,000 deaths as well as great economic burden in farm and pet animals per year [1]. However, its actual prevalence might be still largely underestimated due to a lack of convenient and effective diagnostic approach resulting in underreporting and low awareness in medical and public health communities. The symptom of leptospirosis ranges from an asymptomatic or mild infection to severe manifestation causing multi-organ dysfunction and even deaths in humans [2, 3]. Humans and animals can be infected through the direct or indirect exposure to urine of infected animals and urine-contaminated water or soil [2, 4, 5].

Nowadays, the classical taxonomy typing method of *Leptospira spp*. is mainly based on serological techniques including microscopic agglutination test (MAT) and cross-agglutinin absorption test (CAAT). It is the practical taxon at the subspecies level and remains extremely valuable for epidemiology analysis of *Leptospira*. However, MAT or CAAT is laborious and time-consuming because these methods require the maintenance of a large range of reference strains and corresponding rabbit antisera. In addition, some serovars were found to have a across reaction. Therefore, MAT or CAAT is no longer sufficient to identify isolates to their species level. Recently, several molecular typing methods have been developed to discriminate *Leptospira spp* including PCR-restriction endonuclease analysis, pulsed-field gel electrophoresis (PFGE) [6–8], multilocus variable number of tandem repeats analysis (MLVA) [9, 10]. The most commonly used multilocus sequence typing (MLST) scheme has been recommended as a routine typing *Leptospira* species method and population phylogenetic analysis [11–14].

To date, *Leptospira* genus is now classified into 9 pathogenic, 5 Intermediate and 6 saprophytic species [11, 15, 16]. *L*. *interrogans*, *L*. *borgpetersenii* and *L*. *kirschneri* are the main pathogenic species of leptospirosis in humans and animals worldwide. Based on antigenic similarity, more than 300 antigenically related pathogenic serovars are clustered into 24 serogroups in the world, and 75 serovars belonging to 18 serogroups are reported in China. Among them, serogroup Icterohaemorrhagiae is the most predominant epidemic-causing strain in China, and is responsible for more than 60% reported cases of lepotospirosis [17]. However, to date, there is very limited information of the detailed predominant serovars or genotypes of serogroup Icterohaemorrhagiae in China, which plays a crucial role in the epidemiology of leptospirosis. Understanding this role may allow for the development of better control strategies of this disease. The aim of this work was to investigate the genetic diversity of predominantly epidemic serogroup icterohaemorrhagiae of pathogenic *Leptospira* in Mainland China. Therefore, we investigated the genetic characteristics of 120 serogroup Icterohaemorrhagiae strains isolated from leptospirosis patients or rodent sources in five Chinese provinces with the highest leptospirosis prevalence during the past 50 years by a combination of 16S rRNA sequencing and MLST. Our results could provide a more comprehensive overview of the predominant epidemic serogroup icterohaemorrhagiae in Mainland China and should contribute to understanding the changing epidemiological and evolutionary trends of this serogroup. To obtain a more overview of global population structure and microevolution of serogroup Icterohaemorrhagiae *Leptospira* strains, the available MLST data from MLST database and some previous studies described from other countries representing the international strains were introduced and further analyzed. The results in this study may be used as markers to trace pathogenic strains isolated from the environment and host in the near future, as well as to obtain a more complete overview of global population structure and microevolution of *L*. *interrogans* serogroup Icterohaemorrhagiae strains.

## Materials and Methods

### Ethics statement

The information of these patients with leptospirosis in this study was anonymously obtained from national infectious disease surveillance system in China; only lots of the patients in the recent years were required to provide brief informed consent before blood sampling. All of the protocols in the study including collection and application of these anonymous serum specimens were conducted with approval by the ethical committee of the Chinese Center for Disease Control and Prevention (China CDC, Beijing, China).

### *Leptospira* strains, cultivation, chromosomal DNA preparation and serogroup identification

A total of 128 non-epidemiologically related leptospiral isolates, including 120 Chinese strains isolated from five provinces and 8 international reference strains from seven countries (Indonesia, Congo, Denmark, Japan, Zaire, Sri Lanka and Belgium) were used (S1 Table). The 120 Chinese strains were collected from human or rats over a 50 year period (1958~2008). The 8 reference strains (56101, 56102, 56103, 56104, 56108, 56166, 56229 and 56233) were isolated between 1915~1966 (except a Japanese strain without detailed information). Serogroup identification of these leptospiral strains was carried out by MAT with 15 Chinese standard serogroup-specific rabbit antisera from the National Institutes of Food and Drug Control, China, representing the most predominantly pathogenic serogroups of *Leptospira spp*. in China. The serogroup scoring the highest MAT titer of the test stains agglutinating 50% of live leptospiral against a given serogroup-specific rabbit antisera is defined as the presumptive corresponding one. All of the 128 strains were maintained by the National Institute for Communicable Disease Control and Prevention, China. Leptospires were stored long-term at −70°C and have been passaged every six months. When needed, they were subcultured at 30°C in 10ml Ellinghausen-McCullough-Johnson-Harris (EMJH) liquid medium to stationary phase, and genomic DNA was extracted using NucleoSpin Tissue kits (Macherey-Nagel, Germany) according to the manufacturer’s protocol.

### Species identification

As a reference method of species identification, 16S rRNA gene sequencing was performed as previously described by Morey [18] for all the 128 epidemic strains. A total of 20 accessible *Leptospira* species reference sequences that represented pathogenic, intermediate pathogenic and non-pathogenic *Leptospira* species were obtained from GenBank database and *Turneriella parva* NCTC 11395T and *Leptonema illini* NCTC 11301T were set as outgroup (S2 Table) [16, 18]. The sequences of all the *Leptospira* strains in this study and the 20 representative sequences from GenBank were compared using ClustalW multiple alignments. A Neighbor-joining tree was constructed with Mega software version 5.10 with a bootstrap value of 1,000.

### Molecular typing analysis

MLST were performed based on 7 housekeeping genes including *glmU*, *pntA*, *sucA*, *tpiA*, *pfkB*, *mreA* and *caiB* as previously described [19]. PCR was conducted using the following parameters: an initial denature step at 94°C for 5 min, followed by 30 cycles of 94°C for 30 seconds, 46°C for 30 seconds, 72°C for 45 seconds, then 72°C for 10 min. The PCR products were sequenced by ABI PRISM 377 DNA sequencer. Each allele and the allelic profiles (glum-pntA-sucA-tpiA-pfkB-mreA-caiB) were submitted to the established internet *Leptospira* database (http://leptospira.mlst.net) to assign the sequence types (STs). eBURST algorithm (http://eburst.mlst.net/) was applied to determine the relationships among STs. Clonal complexes (CCs) were defined as multiple STs linked through single locus variants (SLVs) when they differed from each other at a single locus and named on the basis of the putative founder ST or the ST associated with the largest number of SLVs in the clonal complex. Singletons are defined as the STs differing at least three alleles from other STs. Phylogenetic analysis were performed using UPGMA by the BioNumerics software version 5.1 (Applied Maths, Kortrijk, Belgium). Furthermore, multiple concatenated sequences of 7 housekeeping alleles were performed using CLUSTALW software and Phylogenetic analysis was conducted with MEGA 5.10 [20]. The Neighbor-joining tree was constructed using bootstrapping at 1,000 bootstrap replications. To explore the genetic diversity and evolutionary relationship between the isolates in China and other countries, 121 international isolates previously identified by MLST were added into our analysis (S3 Table) [21–23]. Among them, a total of 19 international strains belonging to serogroup Icterohaemorrhagiae from 10 countries, including 5 Chinese isolates in present study, were downloaded from the *Leptospira* MLST website. In addition, 102 sequence data related to Brazil, Argentina and Russia were obtained from three previous studies [21–23]. All of the 121 international strains are listed in S2 Table. The genetic relationship among the 128 isolates in our lab and the 116 isolates from MLST database and previous studies were further analyzed by a minimum spanning tree (MST) analysis using the BioNumerics software version 5.1 (Applied Maths, Inc., Austin, TX, USA).

## Results

### Serogroup identification

All the 128 strains with the highest agglutinating MAT titer against serogroup Icterohaemorrhagiae of 15 standard serogroup-specific rabbit antisera were confirmed as serogroup Icterohaemorrhagiae in this study.

### Species identification using 16S rRNA sequencing

**Fig 1:**
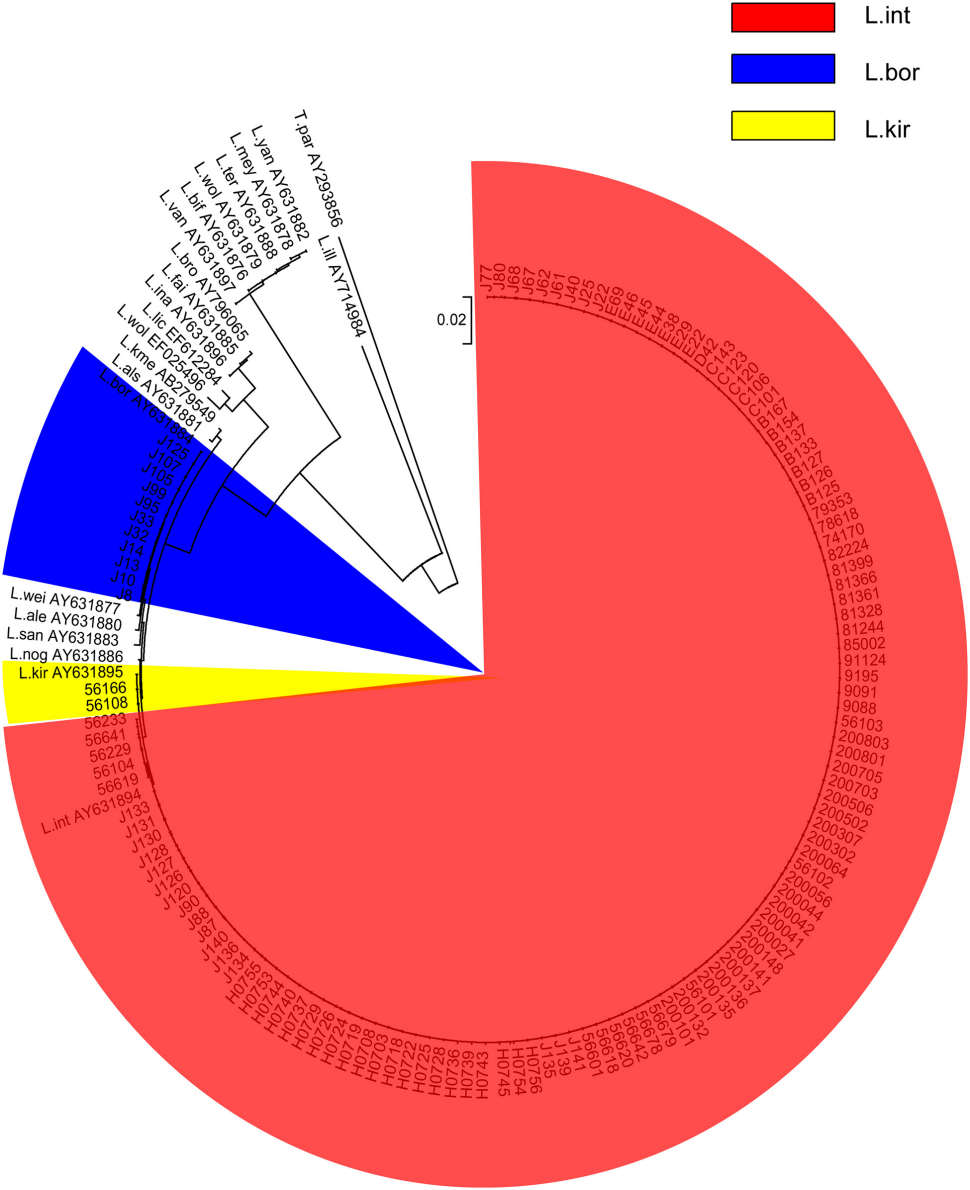
Phylogenetic analysis based on the nearly full-length *rrs* gene for the 128 pathogenic *Leptospira* strains. The evolutionary analysis for the 128 pathogenic isolates was conducted in MEGA5 and evolutionary history was inferred using the Neighbor-Joining method (Tamura, Peterson, Peterson *et al*., 2011) and a bootstrap value of 1000. Twenty reference sequences representing 20 *Leptospira* species originated from GenBank are shown by denoting their species group: pathogenic, intermediate pathogenic, nonpathogenic. *Turneriella parva* NCTC 11395T (AY293856) and *Leptonema illini* NCTC 11301T (AY714984) were considered as outgroup. The scale bar represents the number of base pairs differences. Each species is labeled as follows: abbreviation of species name (L.int: *L*. *interrogans*, L.kir: *L*. *kirschneri*, L.bor: *L*. *borgpetersenii*, L.als: *L*. *alstonii*, L.san: *L*. *santarosai*, L.nog: *L*. *noguchii*, L.wei: *L*. *weilii*, L.ale: *L*. *alexanderi*, L.kme: *L*. *kmetyi*, L.ina: *L*. *inadai*, L.bro: *L*. *broomii*, L.wol: *L*. *wolffii*, L.lic: *L*. *licerasiae*, L.fai: *L*. *fainei*, L.bif: *L*. *biflexa*, L.mey: *L*. *meyeri*, L.wol: *L*. *wolbachii*, L.yan: *L*. *yanagawae*, L.ter: *L*. *terpstrae*, L.van: *L*. *vanthielii*, *T*.*par*: *T*. *parva* and L ill: *L*. *illini*). The dendrogram displays that the 128 *Leptospira* strains belonged to three major clusters corresponding to 3 *Leptospira* species in different colors: Red: *L*. *int*; Yellow: *L*. *kri*; Blue: *L*. *bor*.

Among the 128 strains, 115 strains were identified as *L*. *interrogans*, 11 strains as *L*. *borgpetersenii*, and two reference strains isolated from Congo and Zaire as *L*. *kirschneri* (Fig 1 and S1 Table). Neighbor-joining trees were constructed for the 128 leptospiral isolates in this study and 20 international representative strains obtained from GenBank database (Fig 1). Three distinct groups representative of pathogenic, nonpathogenic, and intermediate *Leptospira* species were obtained. *Turneriella parva* NCTC 11395T and *Leptonema illini* NCTC 11301T formed a distinct basal out-group branch. Compared to the 20 representative sequences, 115 including 109 Chinese isolates from the five provinces (Jiangxi, Sichuan, Anhui, Hunan and Anhui) and 6 international strains isolated from five countries (Belgium, Denmark, Indonesia, Japan and Sri Lanka) were identified as pathogenic *L*. *interrogans*. Eleven isolates identified as the pathogenic *L*. *borgpetersenii* originated from Jiangxi province in China, and the remaining 2 strains isolated from Congo and Zaire were identified as the pathogenic *L*. *kirschneri*.

### Genetic diversity of 128 serogroup Icterohaemorrhagiae isolates using MLST analysis

All of the 128 isolates were successfully amplified and sequenced (S1 Table). The discriminatory ability for different species ranged from 0.11 ST per isolate for *L*. *interrogans* to 1.0 ST per isolate for *L*. *kirschneri* (Table 1).

**Table 1:** Numbers of alleles and sequence types (STs) of 128 pathogenic *Leptospira* strains.

Species	No. of isolates	No. of unique alleles at each locus							No. of STs	No. of STs per isolate
		*glmU*	*pntA*	*sucA*	*tpiA*	*pfkB*	*mreA*	*caiB*		
*L*. *interrogans*	115	2	3	6	5	8	7	5	13	0.11
*L*. *borgpetersenii*	11	1	1	1	1	1	2	1	2	0.18
*L*. *kirschneri*	2	2	1	2	1	1	1	1	2	1.00
Total	128	5	5	9	7	10	10	7	17	0.13

Among 120 Chinese *Leptospira* strains, a total of 10 different STs were obtained, 5 of which were represented by multiple strains, while the remaining 5 STs were found as singleton (Table 2 and S1 Table). The most predominant ST in China was ST1 (69/120), followed by ST128 (18/120), ST17 (17/120), ST143 (9/120), ST209 (2/120) and the remaining 5 isolates belonged to 5 different STs, respectively (Table 2). The most predominant genotype, ST1, was temporally (between 1958 and 2008) and geographically diverse (4 provinces distributed in Sichuan, Jiangxi, Anhui, Hunan). Furthermore, the distributions of STs in China were associated with special geographic regions. For example, ST17, the second most frequent serotype, was found in Sichuan and Jiangxi provinces between 1969~2008 and ST128 was just found in Hunan province in 2007. In addition, ST143 and ST209 were found in Jiangxi province between 2005~2007. It was interesting that only ST143 and ST209 corresponded to *L*. *borgpetersenii* and all the other STs corresponded to *L*. *interrogans* in China. Whereas eight different STs were identified among 8 international strains, only ST17 was found in Chinese *Leptospira* isolates (Table 2 and S1 Table).

**Table 2:** Isolated location and STs of 128 pathogenic *Leptospira* strains.

Strains type	Province of isolation	No. of STs																	Total no. of strains
		ST1	ST17	ST98	ST203	ST128	ST143	ST209	ST92	ST201	ST202	ST199	ST23	ST19	ST141	ST200	ST65	ST38	
**Chinese strains**	**Sichuan**	**26**	**12**	**1**	**1**						**1**								**41**
	**Anhui**	**20**																	**20**
	**Hunan**	**3**				**18**													**21**
	**Jiangxi**	**20**	**5**				**9**	**2**											**36**
	**Yunnan**								**1**	**1**									**2**
**Total Chinese strains**		**69**	**17**	**1**	**1**	**18**	**9**	**2**	**1**	**1**	**1**								**120**
**International Reference strains**	**7 countries**		**1**									**1**	**1**	**1**	**1**	**1**	**1**	**1**	**8**
**Total no. of strains**		**69**	**18**	**1**	**1**	**18**	**9**	**2**	**1**	**1**	**1**	**1**	**1**	**1**	**1**	**1**	**1**	**1**	**128**

### Population structure analysis of 120 Chinese *Leptospira* strains

eBURST analysis based on the allelic profiles was first conducted to identify relationships among 10 *Leptospira* STs found in the 120 Chinese pathogenic strains. Clonal complexes (CCs) based on ST Linkages were built using the criteria of at least five shared alleles. Two CCs (CC1 and CC143) and 5 singletons were identified, including the largest CC1 and the largest singleton CC17 (S1 Fig and S1 Table). The CC1 and 5 singletons belonged to *L*. *interrogans*, whereas CC143 belonged to *L*. *borgpetersenii*. The CC1 contained 69 ST1 strains, 18 ST128 strains and 1 ST98 strain with ST1 being the most likely ancestral strain of this CC. The CC143, including 9 ST143 strains and 2 ST209 strains, showed no predicted founder type. eBURST analysis has confirmed that there is no coexistence of different species within the same CCs.

**Fig 2:**
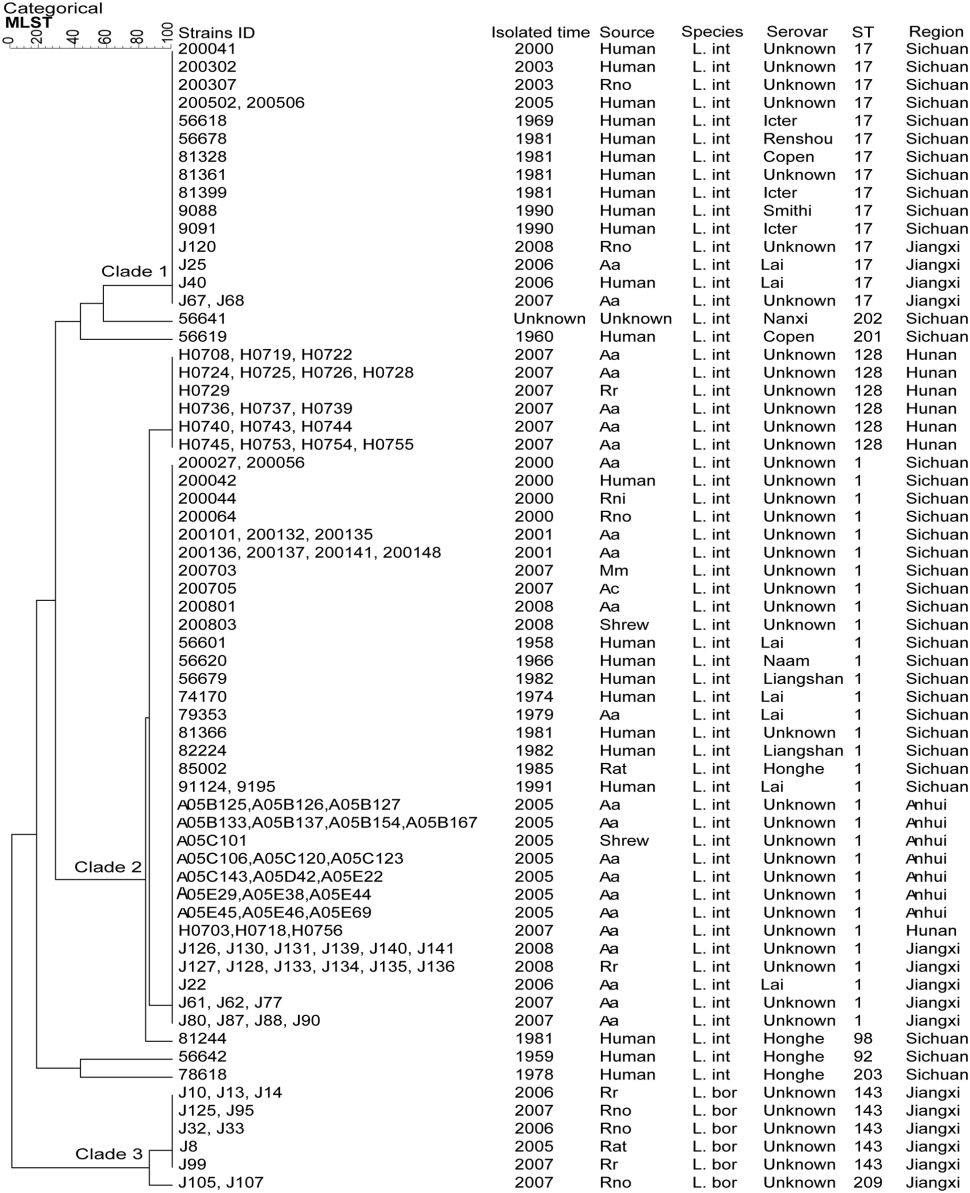
UPGMA dendrogram indicating the similarities of 120 Chinese *Leptospira* strains determined by seven gene loci in MLST. Groups were defined by similarity of 80%. The dendrogram displays that the 120 Chinese *Leptospira* strains belonged to three major clades (Clade1-3) and the remaining isolates were dispersed as unrelated singletons. (L.int: *L*. *interrogans*, L.kir: *L*. *kirschneri*, L.bor: *L*. *borgpetersenii*,Rno: Rattus norvegicus, Aa: Apodemus agrarius, Rr: Rattus rattoides, Rni: Rattus nitidus, Mm: Micromys minutus, Ac: Apodemus chevrieri, Icter: Icterohaemorrhagiae, Copen: Copenhageni).

On the other hand, the relationships between the 10 STs representing 120 Chinese strains were depicted in a UPGMA dendrogram. Three main clades (Clade1-3) were generated and the remaining isolates were dispersed as unrelated singletons (Fig 2). The UPGMA dendrogram revealed ST clustering patterns relatively similar with eBURST analysis (Fig 2 and S1 Fig). The three clades in UPGMA dendrogram corresponded to the CC1, CC143 and one Singleton CC17 in eBURST dendrogram, respectively. The rest of the strains were dispersed as unrelated singletons like the ones in eBURST dendrogram. The Clade2 corresponding to CC1 was found among four provinces (Sichuan, Jiangxi, Hunan and Anhui) and no relationship was observed between the isolates. Furthermore, the UPGMA dendrogram had shown a geographical relationship between the isolates and STs. For instance, The Clade3 corresponding to CC143 contained 11 ST143 and 2 ST209 strains isolated from Jiangxi province, The Clade1 corresponding to singleton CC17 contained 17 ST17 strains from Sichuan and Jiangxi provinces between 1969~2008 and one SLV of ST128 in Clade2 contained 18 strains isolated from Hunan province.

### Global population structure analysis of 244 international *Leptospira* isolates

Besides the 128 strains in this study, additional 116 serogroup Icterohaemorrhagiae isolates with diverse geographical regions or countries from MLST database and other studies were also added to perform MLST analysis. However, among the finally identified 22 STs from these 244 strains, only ten STs were found in China. The eBURST analysis revealed five CCs and 8 singletons (S2 Fig). CC17 remained to be the most predominate CC which covered 125 strains corresponding to three different STs (ST17, ST199 and ST206) and followed by the CC1 including 89 strains corresponding to another three STs (ST1, ST128 and ST98). The third largest CC143 included 11 ST143 strains and 2 ST209 strains isolated from China. ST1 and ST17 were defined as the predicted founders of CC1 and CC17, respectively. The remaining three CCs comprised relatively dispersed STs with no predicted founding type. For instance, CC38 included two relatively distant STs: ST203 and ST38.

The geographic distribution and corresponding STs among the 244 international *Leptospira* isolates are listed in Table 3. Generally close clustering of these strains from same geographical regions was observed. For example, 9 (ST1, ST92, ST98, ST128, ST143, ST209 ST201, ST202 and ST203) of 10 STs were found exclusively in China between 1958~2008. And all of the Brazil, Argentina and Belgium isolates belonged to ST17, all nine Russia isolates and two Denmark isolates were clustered together into CC17. The remaining isolates from Japan, Malaysia, Sri Lanka, and Indonesia were classified as relatively independent singletons. Therefore, ST17 was found as one of the most common STs worldwide, including in Asia (China, Japan), Latin America (Brazil and Argentina) and Europe (Denmark, Belgium and Russia) between 1915~2009.

**Table 3:** Geographic distribution and STs of the 244 Chinese and international *Leptospira* isolates.

Continent	Country	No. of strains	No. of STs	Mainly STs	STs	Strains origin
Asia	China	126	10	ST1,ST128,ST17,ST143,ST209	ST1,ST128,ST17,ST143,ST209,ST92,ST98,ST201,ST202,ST203	This study and MLST database
Asia	Japan	2	2	ST17,ST200	ST17,ST200	This study and MLST database
Asia	Malaysia	2	2	ST21,ST28	ST21,ST28	MLST database
Asia	Sri Lanka	2	1	ST38	ST38	This study and MLST database
Asia	Indonesia	4	2	ST19,ST23	ST19,ST23	This study and MLST database
Africa	Zaire	4	3	ST65	ST65,ST122,ST127	This study and MLST database
Africa	Congo	1	1	ST141	ST141	This study
Latin America	Jamaica	1	1	ST122	ST122	MLST database
Latin America	Brazil	91	1	ST17	ST17	MLST database and Ref.23
Latin America	Argentina	3	1	ST17	ST17	Ref.21
Europe	Denmark	2	2	ST17,ST199	ST17,ST199	This study and MLST database
Europe	Belgium	2	1	ST17	ST17	This study and MLST database
Europe	Russia	9	3	ST17	ST17,ST199,ST206	Ref.22

**Fig 3:**
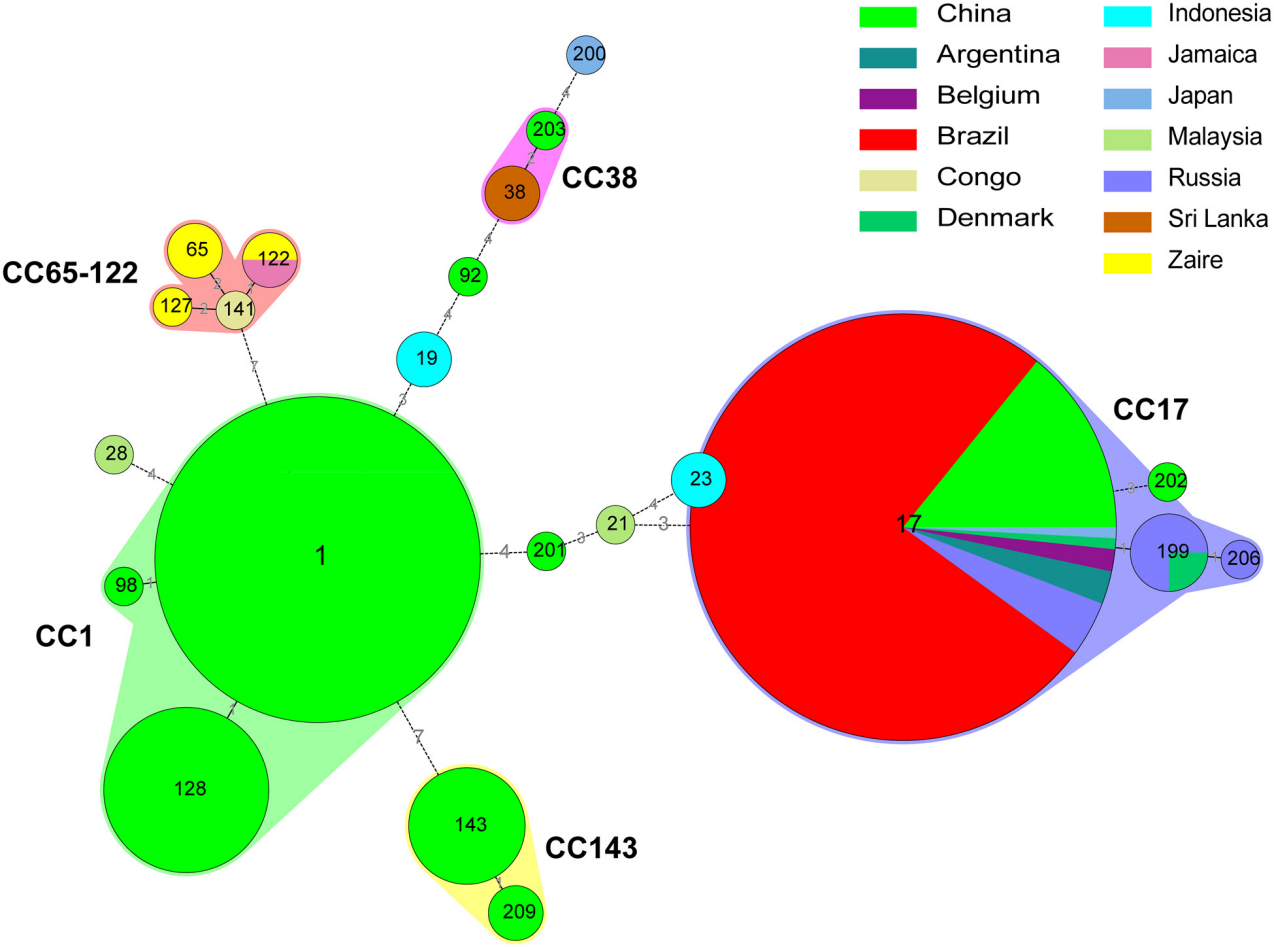
Minimum spanning tree analysis of the 22 STs representing 244 global *Leptospira* strains. In the Minimum Spanning Tree (MST) analysis, each circle represents a different sequence type (ST) as labeled, its size is proportional to the number of strains and the color indicates the country of origin. The width of the lines reflects the genetic difference between two STs, where in dark/heavy lines connect single locus variants (SLVs) or double locus variants (DLVs) and dotted lines indicate the most likely connection between two STs differing by three or more loci.

At the same time, the 244 strains were further analyzed by minimum spanning tree (MST). Five CCs (CC1, CC17, CC143, CC38 and CC65-122) were generated and the remaining isolates were dispersed as unrelated singletons (Fig 3). The MST dendrogram showed relatively similar ST clustering patterns with eBURST analysis (Fig 3 and S2 Fig). In some cases, isolates within same CCs were generally restricted to one or several countries. For instance, the CC1 and CC143 only contained these Chinese strains which had closely genetic relationship but were distant from all other isolates, whereas, the other three CCs included isolates from more than one country. For example, The CC17 included 120 clustered isolates from Asia (China, Japan), Latin America (Brazil and Argentina) and Europe (Denmark, Belgium and Russia). CC38 included 3 clustered isolates from China and Sri Lanka. The CC65-122 included 6 clustered isolates from Africa (Zaire, Congo) and Latin America (Jamaica) corresponding 4 different STs. The remaining isolates were dispersed as singletons. Based on the MST dendrogram, more than half of Chinese strains were clustered into three large CCs: CC1 (88/120), CC143 (11/120) and CC17 (17/120). The remaining 4 isolates from China were dispersed as 4 independently singletons. As seen in Table 3 and Fig 3, the genetic diversity of *Leptospira* strains belonging to serogroup Icterohaemorrhagiae from China was generally different from that of isolates elsewhere. From the global population, no common CCs with potential founders were identified as a whole distribution, indicating high diversity of STs.

### Comparison of phylogenies based on MLST versus 16S rRNA gene

**Fig 4:**
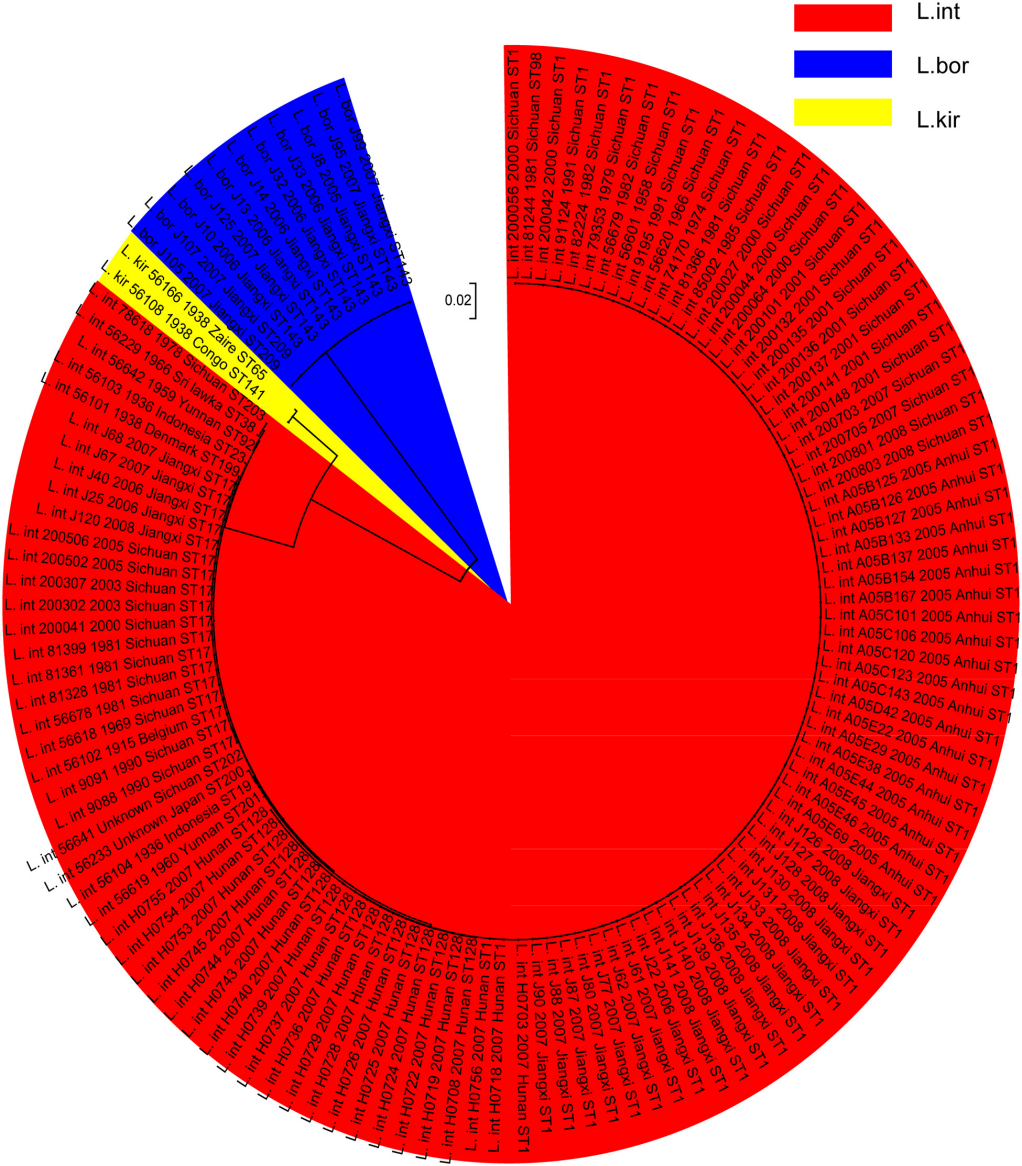
Molecular phylogenetic analysis between 17 sequence types (STs) of 128 pathogenic *Leptospira* strains based on neighbor-joining (N-J) tree method. Phylogenetic relationships based on concatenated sequences of 7-locus MLST scheme (3,102 bp) for the 128 pathogenic strains were inferred using N-J method and 1000 bootstrap replications as implemented in MEGA5. Each bacterial strain is labeled as follows: abbreviation of species name (L.int: *L*. *interrogans*, L.kir: *L*. *kirschneri* and L. bor: *L*. *borgpetersenii*), strain name, isolated time, isolated region and (for the 7-locus MLST scheme) sequence type (ST). The dendrogram displays three major clusters corresponding to 3 *Leptospira* species analyzed in different colors: Red: *L*. *int*; Yellow: *L*. *kri*; Blue: *L*. *bor*.

Based on seven MLST housekeeping genes, Neighbor-joining trees were constructed with three distinct clusters corresponding to three different *Leptospira* species (Fig 4). The *L*. *interrogans* cluster containing 6 international strains and 109 Chinese strains. The *L*. *borgpetersenii* cluster containing 11 strains were further divided into two sub-groups that originated from Jiangxi province between 2005~2007. In addition, the *L*. *kirschneri* cluster containing 2 strains originating from Congo. Phylogenetic analysis revealed relatively similar species clustering patterns with 16S rRNA gene sequencing.

### Corresponding relationship between STs and serovar designations

Among the 120 Chinese strains in this study, 31 isolates with previously confirmed serovar information were utilized to investigate the relationships between serovars and STs (S4 Table). It was found that there were some isolates in same STs generally corresponding to two or more different serovars. 12 STs contained strains in a single serovar (S4 Table). However, for the first time, we reported that ST1 strains distributed among 4 serovars: Lai, Naam, Liangshan and Honghe, and similarly ST17 corresponded to serovar Icterohaemorrhagiae, Lai, Copenhageni, Renshou and Smithi. In addition, interestingly, some serovars were also found to correspond to multiple STs. For example, serovar Copenhageni was found among three STs-ST17, ST199 and ST201. Serovar Honghe was also associated with ST1, ST92, ST98 and ST203. Serovar Lai was associated with ST1 and ST17 and serovar Naam was associated with ST1 and ST23. These observations have shown that the relationship between serovars and STs was highly complicated, suggesting serovar classification as a poor indicator of genetic relatedness.

## Discussion

Although the incidence of leptospirosis has significantly decreased in the past few years, leptospirosis is still considered as an important zoonosis in China. Since 2004, leptospirosis was routinely included in the national epidemic surveillance system that included systematic case reporting and the monitoring efforts aimed at environmental and host animal populations such as pigs, dogs, cattle and rats. The southern provinces of Mainland China had the highest leptospirosis prevalence rates in recent years. A recent report indicated that serogroup Icterohaemorrhagiae has been historically the most prevalent serogroup associated with human and animals outbreaks in China [17].

MAT has been performed only in a limited number of reference laboratories, primary due to the requirement of long-term maintenance of large range of reference strains and serogroup or serovar-specific standard anti-rabbit sera. In addition, pathogenic *Leptospira spp*. include more than 230 serovars, the majority of them have no corresponding specific antisera and cannot be identified by MAT. On the contrary, MLST has a higher discriminatory power among *Leptospira spp*. and is widely used for bacterial genotyping [24], including *Leptospira* [12, 13, 19, 25]. 16sRNA sequencing used as a tool for phylogenetic analysis has led to a better understanding of evolution of *Leptospira*. These two techniques can be directly applied to biological (serum, urine or blood of maintenance hosts and human) and environmental samples. Furthermore, MLST is also supported by an updated website at http://leptospira.mlst.net/, which helps to exchange of new information among laboratories or countries. This would allow for epidemiological studies in some laboratories where they are not able to culture *Leptospira spp*. So far, no detailed studies focusing on the major prevalence and the genetic characterization of leptospirosis disease are available. To investigate the genetic diversity of leptospirosis, a total of 120 Chinese strains and 8 international reference strains belonging to serogroup Icterohaemorrhagiae were analyzed using 16S rRNA gene sequencing and MLST analysis. These isolates were primarily obtained from leptospirosis patients, or a wide range of rodent sources from five major provinces known to have a high incidence of leptospirosis in China. All the 120 strains in this study were differentiated effectively as indicated by clustering patterns (Fig 1). Two different pathogenic species of *L*. *interrogans*, *L*. *borgpetersenii* were identified, which was in agreement with previous studies in China [17, 26]. Among the 120 Chinese isolates, *L*. *interrogans* accounted for 90.83% (109/120); this has been the predominant pathogenic species of leptospirosis in China over the last fifty years (1958–2008). These findings were in agreement with previous studies conducted in Guizhou province [27, 28]. 9.17% (11/120) strains isolated in Jiangxi province between 2006~2007 in China were identified as *L*. *borgpetersenii*. One previous report found that Icterohaemorrhagiae was the serogroup in 51 *L*. *interrogans* and *L*. *kirschneri* strains isolated from a variety of sources and geographical areas in France [25]. In addition, 43 *L*. *interrogans* were uncovered in three outbreaks in Brazilian urban centers [29]. Serovar Copenhageni accounted for 87% of *L*. *interrogans* cases in another large urban outbreak in Brazil [30]. The predominant pathogen species isolated in Mayotte were *L*. *borgpetersenii* and *L*. *kirschneri* [31]. Thaipadungpanit et al. in 2007 had demonstrated that ST34, corresponding to *L*. *interrogans* serovar Autumnalis, accounted for 76% of isolates in 101 *L*. *interrogans* isolates in Thailand [12]. Together, these data revealed that the major *Leptospira* species studied here from different counties were distinct and that the great genetic diversity in geographic epidemiology shown by these isolates reflected this observation. [13, 19, 23, 25]. When compared with *L*. *borgpetersenii*, the two species of *L*. *interrogans and L*. *kirschneri* seem to have more closely related to one another and probably evolved from the *L*. *noguchii* clade. Close phylogenetic relationships between *L*. *interrogans*, *L*. *kirschneri* and *L*. *noguchii* were reported by Ahmed et al. based on MLST phylogenetic analysis [13].

Furthermore, the UPGMA dendrogram revealed relatively similar ST clustering patterns with eBURST analysis, 2 CCs (CC1 and CC143) and 5 singletons were clustered in 120 Chinese strains (Fig 2 and S1 Fig). CC1 consisted of 3 different STs (ST1, ST128 and ST98) from diverse sources over the past 50 years in China, with ST1 as the likely founder. Our results were similar with those of previous studies performed in Guizhou province [32]. The predominant serotype in China, ST1, was widely distributed (37.68% (26/69) Sichuan province; 28.99% (20/69) Jiangxi province; 28.99% (20/69) Anhui province and 4.35% (3/69) Hunan province). The host ranges were 68.12% (47/69) in *Apodemus agrarius*, 13.04% (9/69) in human, 13.04% (9/69) in *Rattus rattoides* and 13.04% (9/69) in *Rattus norvegicus* during the 1958~2008 collection time span. In addition, ST17, widely distributed in Sichuan and Jiangxi provinces, was the second most common serotype isolated during this time period. In general, the predominant serotypes, ST1 and ST17, having distinct sources yet formed a tight group, indicating that there might be one original strain which subsequently diverged evolutionarily into the two STs above within southern China provinces. The remaining 4 singletons of ST92, ST201, ST202 and ST203 shared no more than 3 strains, and presumably dispersed independently. The genotyping results from this study showed that *Apodemus agrarius* could be a main source of leptospirosis transmission in China. MLST is also useful to explore the transmission of specific species between maintenance animal hosts and human. Therefore, it may be useful to implement control strategies for *Apodemus agrarius* to reduce the transmission from animals to humans.

Interestingly, although serogroup Icterohaemorrhagiae strains were found in most *Leptospira* endemic regions in China and some STs such as ST1/ST17 were widely distributed in this study, there were some prominent serogroups consisting of more than one ST/species in specific regions. For example, CC143, belonging to *L*. *borgpetersenii*, comprised of 11 strains isolated from *Rattus rattoides* and *Rattus norvegicus*. CC143 was locally confined to Jiangxi province between 2006~2007 in China. One SLV of ST128 comprised of 18 strains belonging to *L*. *interrogans*, and was isolated from *Apodemus agrarius* and *Rattus rattoides* hosts in Hunan province in 2007. Therefore, some clustering of strains from specific geographical regions was observed in China. The strains isolated from Jiangxi and Hunan provinces were from the same monitoring sites, respectively, suggesting that the isolates may have an epidemiological link in that given locale. This also gives a clue that the MLST scheme is capable of dissecting the molecular geographic epidemiology of leptospirosis. No other obvious epidemiological relationship was found between STs and source specimens or isolated locations in these 120 Chinese strains. These results were not surprising because these isolates were epidemiologically unrelated and showed a great diversity in STs; no clustering was detected. More isolates and molecular typing data are needed in order to better understand the epidemiology of leptospirosis in China. Basis on the genotyping results in this study, certain *Leptospira* genotypes are prevalent in a particular geographical region and associated with special animal reservoirs. The diverse distributions of genotypes may provide a clue for species-specific vaccine preparation to increase the efficacy of a vaccination program in different epidemic regions. This information may also be useful for tracking of the source of leptospirosis outbreak and to establish a control program against leptospirosis in each region.

In order to explore the global genetic diversity and evolutionary relationships in the serogroup Icterohaemorrhagiae strains worldwide, a total of 244 serogroup Icterohaemorrhagiae strains from 13 different countries were analyzed and 22 STs were found. The MST dendrogram revealed relatively similar ST clustering patterns with eBURST analysis; five CCs (CC1, CC17, CC38, CC143 and CC65-122) and 8 singletons were clustered in 244 international strains (Fig 3 and S2 Fig). CC1 and CC143 were the dominant clones in China; these two CCs shared a close genetic relationship and were distant from all the other global isolates. The other 3 CCs, on the other hand, included isolates from more than one country. The predominant ST recovered in Asia, Latin America and Europe between 1915~2009 was ST17. Furthermore, the remaining isolates were dispersed in 8 unrelated singletons. In addition, the eBURST and MLST analyses revealed that the genetically diverse species/strains of serogroup Icterohaemorrhagiae isolates from China was generally different from those isolated in other countries belonging to that particular serogroup. The remaining 9 STs were found in China exclusively, which may indicate that *Leptospira* may evolve according to different locations and the epidemiology of leptospirosis in China is relatively independent from other countries. This also indicated that MLST is a useful technique to explore the genetic diversity and molecular epidemiology of leptospirosis on a global and/or historical scale.

What is more, Thaipadungpanit et al. had applied MLST typing scheme to 101 *L*. *interrogans* isolates and 12 STs were identified in Thailand in 2007. Among the 12 STs found, ST34, corresponding to *L*. *interrogans* serovar Autumnalis, accounted for 76% of isolates [12]. Moreover, Caimi et al. demonstrated that ST37 corresponded to two serogroups of Pomona and Canicola, and was the most frequent genotype in 18 isolates in Argentina. All the 3 serogroup Icterohaemorrhagiae strains isolated between 1993~2005 were identified as ST17 [21]. Among 11 serogroup Icterohaemorrhagiae strains in Russia, four STs (ST17, ST199, ST23 and ST206) were found [22]. It was previously reported that Icterohaemorrhagiae was the most prevalent serogroup in Brazil [23, 33], and all the 90 serogroup Icterohaemorrhagiae strains isolated between 1986~2009 in the state of Sao Paulo were identified as ST17 [23]. In all, it was indicated that the predominant serogroups or STs were different in different geographical regions of the world. Whereas, ST17 was the most predominant ST in serogroup Icterohaemorrhagiae in Argentina, Russia and Brazil and ST1 was the most frequent ST in serogroup Icterohaemorrhagiae in China irrespective of the scattered spatial and geographic distribution. These predominant isolates are likely to have adaptive selective advantages in the environment or in maintenance hosts, allowing them to develop into pathogenic strains.

Based on concatenated sequences of the 7-locus MLST scheme, 128 strains were differentiated effectively into three distinct clusters corresponding to three species, *L*. *interrogans*, *L*. *kirschneri* and *L*. *borgpetersenii* (Fig 2) by Phylogenetic analysis. This is consistent with previous studies that MLST allowed differentiation of the major pathogenic species of *Leptospira* [13, 19, 23, 25]. The Neighbor-joining tree revealed the phylogenetic relationship between these three different pathogenic species in this study and had shown that two pathogenic species of *L*. *interrogans* and *L*. *kirschneri* seem to be more closely related than *L*. *borgpetersenii*, which was also confirmed using 16S rRNA sequencing in this study. The close genetic relationship of *L*. *interrogans* and *L*. *kirschneri* was also confirmed by Boonsilp et al [11]. From the Phylogenetic analysis of MLST data, the *Leptospira* strains belonging to the same serovars were not clustered together. This was also confirmed in earlier findings [12, 13, 19, 23]. This may be due to horizontal gene transfer. Therefore, the MLST method is an alternative suitable method to identify *Leptospira* up to genome species level.

To explore the relationship between STs and serovars, 31 isolates that had both STs and serovar designations in this study were analyzed. We found that there were some isolates belonged to the same STs, but generally corresponded to different serovars. On the other hand some serovars usually were associated with more than one different ST. These observations have shown that serovars are not suitable indicators of genetic relatedness. The diversity of serovars is most likely to be due to horizontal gene transfer events, leading to differences in sequences.

Here our focusing on serogroup Icterohaemorrhagiae strains of *Leptospira* using MLST analysis and 16sRNA gene sequencing as a tool for phylogenetic analysis has led to a better understanding of evolution of *Leptospira*. MLST provides evidence that the diversity of STs among the serogroup Icterohaemorrhagiae strains is very high in China. The result may be useful to develop a strategy and/or guidelines for the control of leptospirosis in China. However, phylogenetic analysis of more globally dispersed *Leptospira* strains is necessary; we nonetheless believe that our present study provides a blueprint for further phylogenetic research. More convenient molecular techniques have to be developed to identify and characterize *Leptospira* species and STs.

## Supporting Information

S1 Fig eBURST diagram of relationships between 10 *Leptospira spp*. sequence types (STs) among 120 Chinese isolates. Clonal complexes (CCs) were built based on ST linkages by TLV criteria. Representation of the 2 CCs and 5 singletons of *Leptospira* spp were found. The size of each dot is proportional to the number of strains in each ST. STs assigned to the same CC are linked by straight lines.
(TIF)

S2 Fig eBURST diagram of relationships between 22 *Leptospira* spp sequence types (STs) among 244 global worldwide isolates. Representation of the 5 CCs and 8 singletons of *Leptospira* spp were found. The size of each dot is proportional to the number of strains in each ST. STs assigned to the same CC are linked by straight lines.
(TIF)

S1 Table 128 pathogenic *Leptospira* strains used in this study. (XLS)

S2 Table 16S rRNA gene sequences of 20 *Leptospira* reference species, *Turneriella parva* NCTC 11395T and *Leptonema illini* NCTC 11301T obtained from GenBank database. (DOC)

S3 Table 121 international *Leptospira* strains belonging to serogroup Icterohaemorrhagiae obtained from MLST database and previous studies described. (XLS)

S4 Table Thirty-one pathogenic *Leptospira* strains with both STs and serovar designations in this study. (XLS)
