# Supplementary figures and images for: Molecular Typing of Pathogenic Leptospira Serogroup Icterohaemorrhagiae Strains Circulating in China during the Past 50 Years
Source: PLoS Negl Trop Dis. 2015 May 19;9(5):e0003762. doi: 10.1371/journal.pntd.0003762 (PMC4437656; doi:10.1371/journal.pntd.0003762)

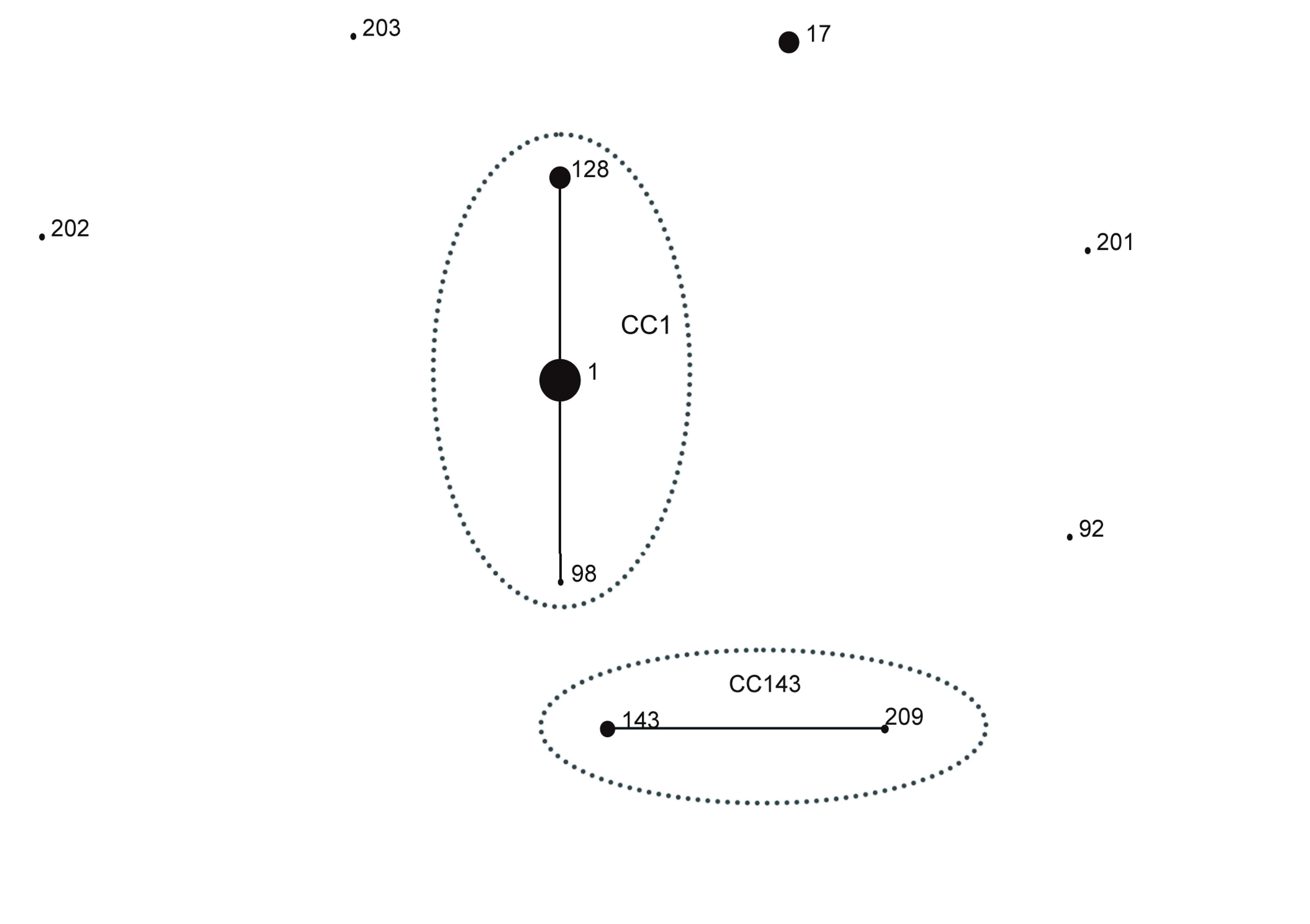

Supplement: S1 Fig — Clonal complexes (CCs) were built based on ST linkages by TLV criteria. Representation of the 2 CCs and 5 singletons of Leptospira spp were found. The size of each dot is proportional to the number of strains in each ST. STs assigned to the same CC are linked by straight lines. (TIF) [file pntd.0003762.s001.tif]

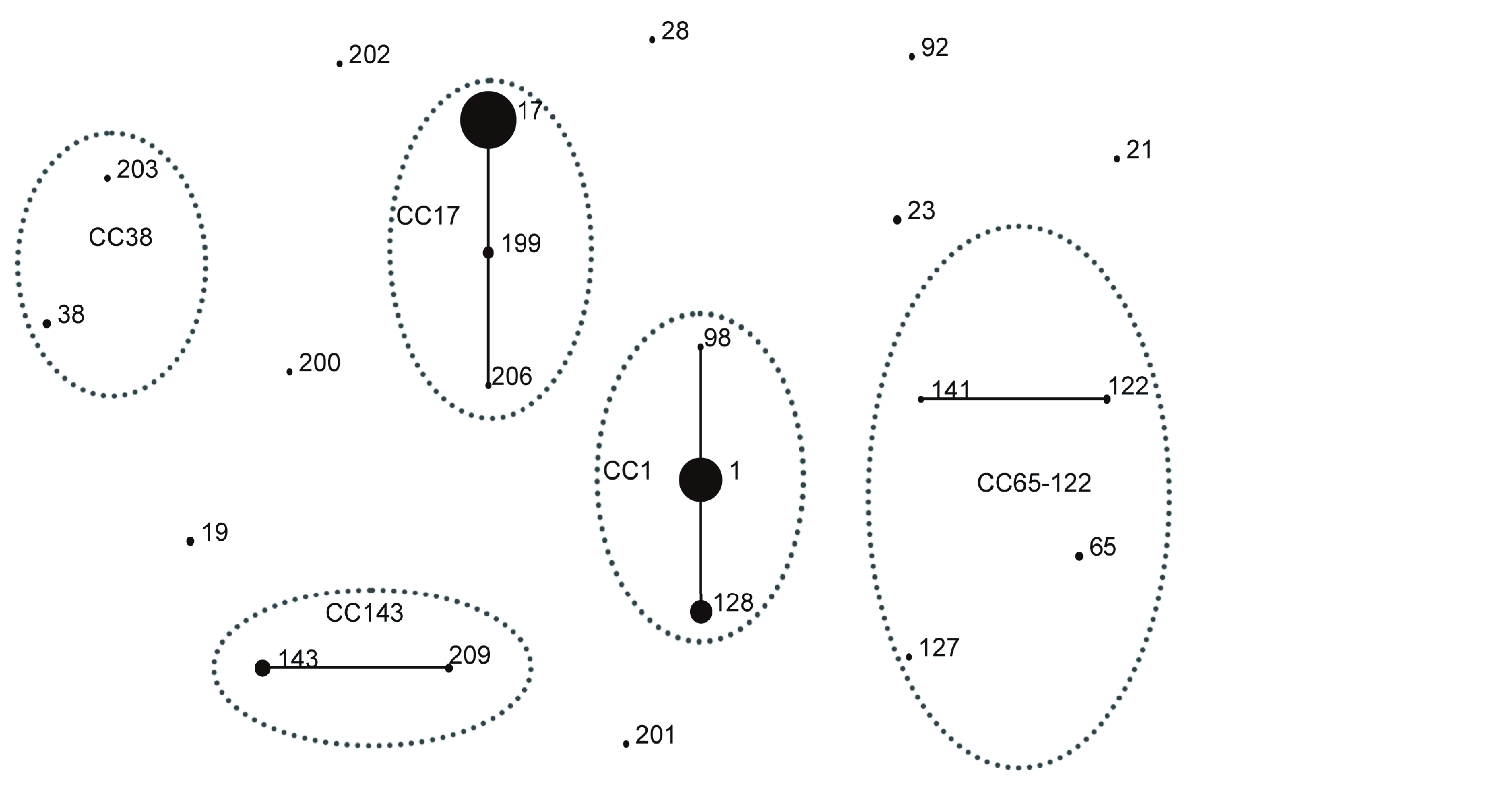

Supplement: S2 Fig — Representation of the 5 CCs and 8 singletons of Leptospira spp were found. The size of each dot is proportional to the number of strains in each ST. STs assigned to the same CC are linked by straight lines. (TIF) [file pntd.0003762.s002.tif]
