# Supplementary material for: Molecular Typing of Pathogenic Leptospira Serogroup Icterohaemorrhagiae Strains Circulating in China during the Past 50 Years
Source: PLoS Negl Trop Dis. 2015 May 19;9(5):e0003762. doi: 10.1371/journal.pntd.0003762 (PMC4437656; doi:10.1371/journal.pntd.0003762)
Supplement: S2 Table — (DOC) [file pntd.0003762.s004.doc]

Table S2. 16S rRNA gene sequences of 20 *Leptospira* species, *Turneriella parva* NCTC 11395T and *Leptonema illini* NCTC 11301T obtained from GenBank database

| Clade | Species | Serovar | Strain | GenBank accession no. |
| --- | --- | --- | --- | --- |
| Pathogenic | *L. alstonii* | Sichuan | ATCC BAA-2439 ^T^ | AY631881 |
| Pathogenic | *L. santarosai* | Shermani | ATCC 43286 ^T^ | AY631883 |
| Pathogenic | *L. borgpetersenii* | Ballum | Mus 127 | AY631884 |
| Pathogenic | *L. noguchii* | Panama | ATCC 43288 ^T^ | AY631886 |
| Pathogenic | *L. interrogans* | Ballum | RGAT | AY631894 |
| Pathogenic | *L. kirschneri* | Cynopteri | ATCC 49945 ^T^ | AY631895 |
| Pathogenic | *L. weilii* | Celledoni | ATCC 43285 ^T^ | AY631877 |
| Pathogenic | *L. alexanderi* | Manhao 3 | ATCC 700520 ^T^ | AY631880 |
| Pathogenic | *L. kmetyi* | Not designated | Bejo Iso9 ^T^ | AB279549 |
| Intermediate | *L. inadai* | Lyme | Lyme 10 ^T^ | AY631896 |
| Intermediate | *L. broomii* | Not designated | ATCC BAA-1107 ^T^ | AY796065 |
| Intermediate | *L. wolffii* | Not designated | Khorat-H2 ^T^ | EF025496 |
| Intermediate | *L. licerasiae* | Not designated | ATCC BAA 1110 ^T^ | EF612284 |
| Intermediate | *L. fainei* | Hurstbridge | ATCC BAA-1107 ^T^ | AY631885 |
| Nonpathogenic | *L. biflexa* | Patoc | ATCC 23582 ^T^ | AY631876 |
| Nonpathogenic | *L. meyeri* | Ranarum | ATCC 43287 ^T^ | AY631878 |
| Nonpathogenic | *L. wolbachii* | Codice | ATCC 43284 ^T^ | AY631879 |
| Nonpathogenic | *L. yanagawae* | Saopaulo | ATCC 700523 ^T^ | AY631882 |
| Nonpathogenic | *L. terpstrae* | Hualin | ATCC 700639 ^T^ | AY631888 |
| Nonpathogenic | *L. vanthielii* | Holland | ATCC 700522 ^T^ | AY631897 |
| Other | *T. parva* | Parva | NCTC 11395 ^T^ | AY293856 |
|  | *L. illini* | Illini | NCTC 11301 ^T^ | AY714984 |
